# Impact of Hyponatremia after Renal Transplantation on Decline of Renal Function, Graft Loss and Patient Survival: A Prospective Cohort Study

**DOI:** 10.3390/nu13092995

**Published:** 2021-08-27

**Authors:** Lena Berchtold, Anja Filzer, Rita Achermann, Vasileios Devetzis, Suzan Dahdal, Marco Bonani, Aurelia Schnyder, Dela Golshayan, Patrizia Amico, Uyen Huynh-Do, Sophie de Seigneux, Spyridon Arampatzis, on behalf of Swiss Transplant Cohort Study Collaborators

**Affiliations:** 1 Service of Nephrology, Department of Internal Medicine Specialties, University Hospital of Geneva, 1205 Geneva, Switzerland; lena.berchtold@hcuge.ch (L.B.); sophie.deseigneux@hcuge.ch (S.d.S.); 2 University Clinic for Nephrology and Hypertension, Inselspital, Bern University Hospital of Bern, 3010 Bern, Switzerland; anja.filzer@gmail.com (A.F.); vasdeve@yahoo.gr (V.D.); suzandahdal@gmail.com (S.D.); Uyen.Huynh-Do@insel.ch (U.H.-D.); 3 Department of Transplant Immunology and Nephrology, University Hospital Basel, 4031 Basel, Switzerland; rita.achermann@usb.ch (R.A.); patrizia.amico@usb.ch (P.A.); 4 Division of Nephrology, University Hospital Zurich, 8091 Zurich, Switzerland; marco.bonani@usz.ch; 5 Clinic for Nephrology and Transplant Medicine, Hospital of St. Gallen, 9007 St. Gallen, Switzerland; aurelia.schnyder@kssg.ch; 6 Centre for Organ Transplantation (CTO), 1011 Lausanne, Switzerland; dela.golshayan@unil.ch

**Keywords:** hyponatremia, kidney transplantation, Swiss Transplant Cohort Study

## Abstract

Background: Hyponatremia is one of the most common electrolyte disorders observed in hospitalized and ambulatory patients. Hyponatremia is associated with increased falls, fractures, prolonged hospitalisation and mortality. The clinical importance of hyponatremia in the renal transplant field is not well established, so the aim of this study was to determine the relationships between hyponatremia and mortality as main outcome and renal function decline and graft loss as secondary outcome among a prospective cohort of renal transplant recipients. Methods: This prospective cohort study included 1315 patients between 1 May 2008 and 31 December 2014. Hyponatremia was defined as sodium concentration below 136 mmol/L at 6 months after transplantation. The main endpoint was mortality. A secondary composite endpoint was also defined as: rapid decline in renal function (≥5 mL/min/1.73 m^2^ drop of the eGFR/year), graft loss or mortality. Results: Mean sodium was 140 ± 3.08 mmol/L. 97 patients displayed hyponatremia with a mean of 132.9 ± 3.05 mmol/L. Hyponatremia at 6 months after transplantation was associated neither with mortality (HR: 1.02; *p* = 0.97, 95% CI: 0.47–2.19), nor with the composite outcome defined as rapid decline in renal function, graft loss or mortality (logrank test *p* = 0.9). Conclusions: Hyponatremia 6 months after transplantation is not associated with mortality in kidney allograft patients.

## 1. Introduction

Hyponatremia, classically defined as a serum sodium concentration of less than 136 mmol/L, is one of the most common electrolyte disorders in hospitalized and ambulatory patients [1,2,3,4]. Hyponatremia may be a novel marker for adverse outcomes, and several studies have shown that hyponatremia is associated with increased falls, fractures, prolonged hospitalisation and mortality risk in the general population [5,6,7,8,9,10,11]. Whether hyponatremia has a direct effect on cardiac contractility, insulin resistance or impaired muscular function is it represents an indirect marker of underlying co-morbidities remains unclear.

In liver transplant candidates, hyponatremia is associated with mortality and low graft survival after liver transplantation [12,13,14]. The clinical importance of hyponatremia is not well established in the renal transplant field. 

The kidneys regulate body water and electrolyte balance [1,15]. After kidney transplantation, electrolyte handling is impaired and the incidence of hyponatremia may be high due to a number of deleterious factors including delayed graft function [16] and transplant rejection episodes [17]. In addition, diuretics [11,18,19] and immunosuppressant drugs [20,21], widely used after transplantation, have a long-lasting influence on electrolyte homeostasis.

To date there is no prospective study reporting on the relationship between hyponatremia and renal graft failure or mortality in stable renal allograft recipients. A retrospective analysis from an Asian cohort of 1786 kidney transplant recipients described significant correlations between early onset hyponatremia and mortality or graft failure [22]. A secondary analysis of a European cross-sectional study in 576 stable renal allograft recipients reported an increased risk of allograft loss but not mortality among those with chronic hyponatremia, before and after adjustment for other confounders.

The current study is a nested project of the multicenter Swiss Transplant Cohort Study (STCS) [23]. Our study aims at contributing prospective data regarding hyponatremia and adverse outcome in renal transplantation by determining the relationships between hyponatremia and mortality, and as secondary outcome renal function decline or graft loss among a cohort of renal transplanted patients within the STCS.

## 2. Materials and Methods

### 2.1. Study Design and Data Collection

#### 2.1.1. Characteristics of the STCS

The STCS prospectively enrolls all solid organ transplant (SOT) recipients undergoing transplantation procedures in Switzerland [23]. Approval of the STCS was provided by the corresponding Ethics Committees, that was approved by the Ethics Committees of all participating centers (Swissethics BASEC project 2018–022394). From all enrolled patients, informed consent was obtained prior to transplantation. The database captures both patient- and graft-specific data. Clinical and laboratory data are continuously collected and entered in the database at the time of transplantation, at 6 and 12 months and yearly thereafter. The STCS Central Data Center performs regular data monitoring and in-depth data quality audits. In this process, all participating centres and all types of transplants are assessed by a thorough review process of randomly sampled patients. The study was approved by the Scientific Committee of the STCS, which granted permission to the investigators to use the data from the STCS.

#### 2.1.2. Patients and Data

All adult recipients of lung, heart, liver, kidney, and kidney-pancreas grafts in Switzerland and enrolled in the STCS between 1 May 2008 and 31 December 2014 were included. For our analysis, only kidney allograft recipients’ transplant data were analysed.

Data were obtained before transplantation, six months and every year post-transplantation. Baseline characteristics, including medical history, co-morbidities and treatment, were selected through STCS database, as well as follow up information as renal function evolution, graft loss and death. The laboratory parameters were primarily gathered from the local centres and then cross-referenced within the STCS database. Follow-ups were considered until the end of 31 December 2015 (censoring). Specific exclusion criteria were primary non-function of the graft, multiple organ recipients (except two kidneys), missing sodium level collected at 6 months and loss to follow up within 6 months.

Hyponatremia was defined as a serum sodium concentration of <136 mmol/L at 6 months after transplantation, the cut-off for the lower end of the normal range. We decided to choose serum concentration at 6months to avoid bias related to post-operative factors and to be sure that patients have reached a stable renal function. We compared allograft recipients with hyponatremia (<136 mmol/L) to those with serum sodium ≥136 mmol/L (subjects without hyponatremia).

#### 2.1.3. Outcomes

The main outcome was mortality. A composite secondary endpoint was defined as: rapid decline in renal function, graft loss or mortality. Rapid decline of renal function was defined as a decrease of ≥5 mL/min/1.73 m^2^ drop of the estimated glomerular filtration rate per year relative to the baseline measurement at 6 months.

#### 2.1.4. Statistical Analysis 

Patients were divided into two groups based on their serum sodium level measured at 6 months after transplantation. Patients were assigned to the low sodium group if their level was lower than 136 mmol/L, otherwise to the control group. To examine whether there was an association between the sodium level and outcome death, death or graft loss, we performed a Cox proportional hazard regression adjusted for covariates gender and age. Schoenfeld residuals were checked visually to verify violations of the proportional hazard assumption. For the secondary combined endpoint rapid decline of renal function, graft loss or death, the HR could not be estimated in a reliable way. The reason is that the event of rapid decline of renal function occurs at a specific time point, e.g., at the yearly follow up measurement; the proportional hazard assumption is violated as can be seen from Kaplan Meier Curve (Figure 4) and estimating a time-dependent HR did not solve the problem. Therefore, we only provide results of the log-rank test of the survival curves. For all three outcomes we performed two sensitivity analyses. In one analysis patients with high sodium level were excluded (*n* = 21). High sodium level was defined as >145 mmol/L. For a second analysis we reassigned the patients to the low sodium group according to the sodium levels measured at 12 months post transplantation. Patients with missing sodium levels (*n* = 37) at 12 months were excluded. 

To investigate the association between sodium level and variables of clinical and biological interest we performed a general linear mixed regression with sodium level and clinical parameters based on previous studies. This analysis included information at 6 and 12 month after transplantation from the STCS or collected additionally in the transplant centre. The statistical significance was determined as a *p* < 0.05, and all tests were two-sided. Statistical analyses were performed using R software (R Foundation for Statistical Computing, Vienna, Austria).

## 3. Results

### 3.1. Baseline Characteristics of Patients

**Figure 1:**
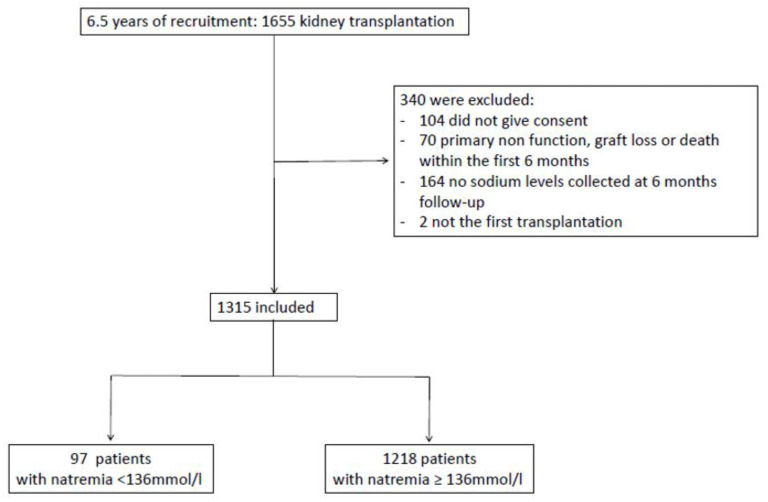
Flowchart illustrating patient recruitment.

From June 2008 to December 2014, a total of 1655 kidney transplantations were performed in the six Swiss centres participating in the STCS Study. We included 1315 kidney transplant recipients (Figure 1). Patients were required to have at least one sodium level collected at 6 months follow up to be eligible. They were mainly male (64.6%) with a median age of 54 (IQR 43-63) years old at transplantation. At 6 months, mean sodium level was 140 ± 3.08 mmol/L. Ninety seven (97) patients displayed hyponatremia (7.4%) and 1218 patients presented sodium ≥136 mmol/L (92.6%). Out of the 97 patients with hyponatremia at 6 months, 24 were still hyponatremic at 12 months (24.7%). Baseline characteristics are presented in Table 1. The median follow-up time of the hyponatremic group was 1465 days (IQR 1085-2189) and 1806 days (IQR 1100-2209) for the patients with sodium levels ≥136 mmol/L. 

This section may be divided by subheadings. It should provide a concise and precise description of the experimental results, their interpretation, as well as the experimental conclusions that can be drawn.

Compared to the patients with ≥136 mmol/L, the hyponatremic group was characterized by a higher rate of pre-transplant hemodialysis and deceased donor organ transplantation, mainly from brain death donors. The groups did not significantly differ in age, sex or comorbidities as hypertension, cardiac insufficiency and liver failure.

### 3.2. Risk Associated with Hyponatremia

The main outcome was mortality. A secondary composite endpoint was defined as: rapid decline in renal function ≥5 mL/min/1.73 m^2^ drop of eGFR per year), graft loss or mortality. During the follow-up period, in the hyponatremic group, four patients died (4.12%), 36 (37.11%) patients had a rapid decline in renal function and three (3.09%) had graft loss. The causes of death were two to palliative care, one liver failure and one cancer. The causes of graft loss were one BK nephropathy, two chronic allograft nephropathies, two immunological disorders and one recurrence of the initial disease. 

At baseline, the Pearson correlation coefficient between sodium and creatinine was not significant (−0.0295, 95% CI −0.07–0.009; *p* = 0.1318). In a mixed linear model, sodium level was associated with glucose, chloride, potassium, albumin level, age, female gender, mycophenolate mofetil and calcineurin inhibitors (Table 2). Association strengths were low apart for high glucose levels and low chloride levels, which were associated with lower natremia. 

**Figure 2:**
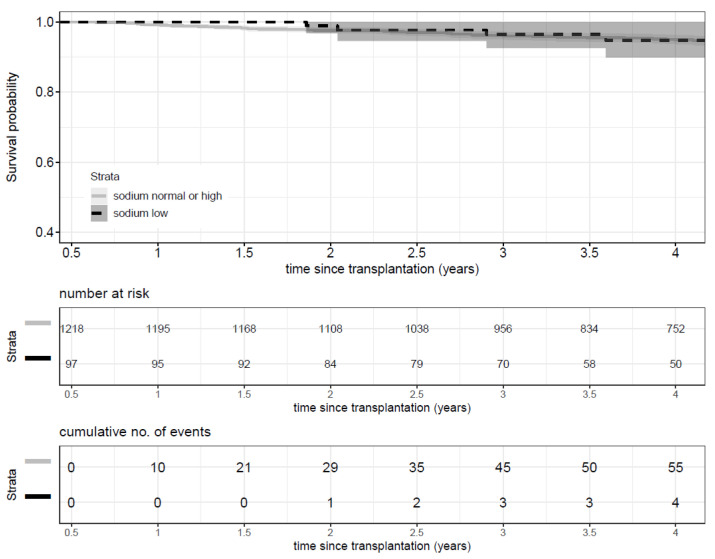
Kaplan Meier curve of outcome of death by sodium level.

Low sodium level at baseline was not associated with an increased risk of death (HR: 1.02; *p* = 0.97, 95% CI: 0.47–2.19). Figure 2 shows the Kaplan-Meier risk curve for the main outcome: mortality.

**Figure 3:**
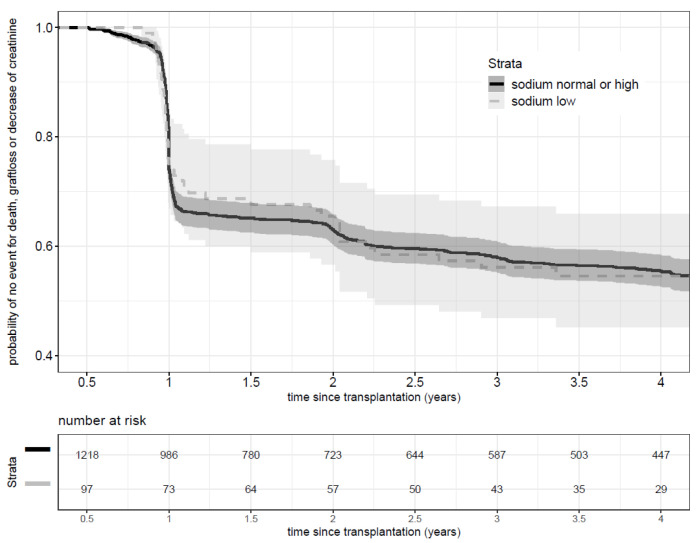
Kaplan Meier curve of composite outcome of death, graft loss or rapid decline of renal function by sodium level.

Hyponatremia at baseline was not associated with rapid decline of renal function, graft loss or death (log rank test *p* = 0.9). Figure 3 shows the Kaplan-Meier risk curves of composite outcome (rapid decline of renal function, graft loss or death). At 15 months, the secondary composite outcome occurred in 30.9% (30/97) of hyponatremic patients, and 34.1% (415/1218) in patients with natremia ≥136 mmol/L. 

**Figure 4:**
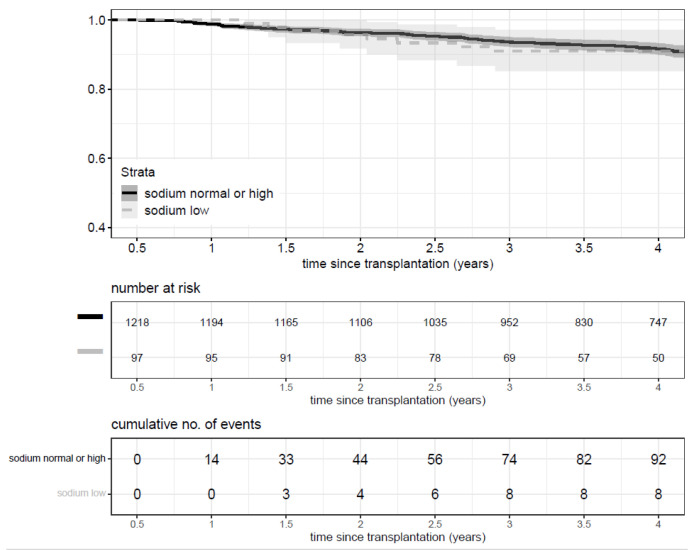
Kaplan Meier curve of outcome of death or graft loss by sodium level.

Hyponatremia was neither associated with graft loss nor with death (HR 1.13, *p* = 0.68, 95% CI: 0.63–2.04) (Figure 4).

We performed sensitivity analyses to examine the impact of serum sodium and relevant outcomes after excluding patients with serum sodium levels >145 mmol/L (*n* = 21). Excluding patients with hypernatremia did not significantly change any of our findings (Supplementary Materials, Table S1). 

We performed another sensitivity analysis to examine the impact of more severe hyponatremia taking a threshold of <132 mmol/L. Among the 18 patients in the hyponatremic group, two died. HR was 2.4 (95% CI 1.02–7.51; *p* value = 0.0464) (Figure S1).

## 4. Discussion

In our cohort study including 1315 stable kidney allograft recipients, we observed a relatively high rate of hyponatremia at 6 months (7.4%). We found no association between sodium level and renal function at baseline. We observed no association between sodium level and use of diuretics. In our cohort, hyponatremia was not associated with the risk of rapid decline of renal function, graft loss or death. Only severe hyponatremia, (defined as serum sodium <132 mmol/L) was associated with death but the number of patients and events in this secondary analysis was very low.

The association between high glucose and low chloride levels with hyponatremia found in our study, likely reflects hypovolemic hyponatremia related to gastro-intestinal losses. Kidney allograft recipients indeed may present some nausea and vomiting related to drug intake. CNIs and other drugs have been shown to have direct tubular effects that could explain these observations. The role of immunosuppressive drugs is supported by their association with hyponatremia. 

The prevalence of hyponatremia in our cohort was quite similar to a recent European study from Vienna, in which hyponatremia was present in 9% of kidney allograft recipients. In this study 2/3 of all allograft recipients showed at least one electrolyte disorder, with hypomagnesemia being the most abundant one [24]. Associated risk factors for development of hyponatremia in this cohort were higher serum potassium and phosphate levels, lower serum magnesium concentrations as well as treatment with ACE inhibitors. 

Our findings are in contrast to the recently published retrospective multicenter cohort study from Korea [22]. In this study, kidney allograft recipients displaying hyponatremia (4%) at 3 months post transplantation had a significantly increased risk of graft failure (HR: 3.21 95% CI 1.47–6.99) and mortality (HR 3.03, 95% CI 1.21–7.54) at follow up. While the cohort size (1480 vs. 1786) and prevalence of hyponatremia were comparable in both studies (6.6% vs. 4%), there are differences in several aspects of methods and cohort characteristics. In contrast to Han et al. [22], we excluded all patients with primary non- function, graft loss or death within the first 6 months post-transplantation. Sodium values were analysed at 6 months, not at 3 months. The fact that early post-transplant complications often lead to electrolyte disturbances could explain why we captured less cases of death and graft loss associated with hyponatremia. Regarding the cohort, mostly Asians were included in Han’s study, the mean age (43 years) was younger than in our cohort (54–56 years) it was mainly living related donors (77.2%) and the use of CNI was lower (59%). Despite the identified association between hyponatremia and poor outcomes in renal transplant recipients by Han et al., a causal link could not be established due to the study design. Moreover, the cause of hyponatremia may be different in Han’s study due to fewer use of CNI. In addition, the frequent postoperative surveillance patterns in Swiss transplant centers along with the current close serum sodium monitoring and the awareness of clinicians concerning hyponatremia may have improved allograft and patient survival.

Few other studies that have investigated the prevalence of hyponatremia and its impact on the outcome of transplant recipients are basically limited to the liver. In patients with end-stage liver-cirrhosis, hyponatremia is associated with a higher rate of complications [25] and a higher mortality when hospitalized or when on the liver-transplant waitlist (TL) [14]. However, regarding the outcome after transplantation, studies do not agree on whether hyponatremia is associated with mortality and graft loss. Dawwas et al. found that low serum sodium contributes to postoperative complications (neurological disorders, infections, kidney failure and longer stay in intensive-care-unit) and higher mortality during the first three months after transplantation [13]. Hackworth et al. also concluded that pre-transplant hyponatremia was associated with a higher complication rate, but they did not find a difference in mortality 180 days after orthotropic liver transplantation [12]. Other studies did not ascertain the relationship between hyponatremia and mortality after 3 months [26,27]. It has to be pointed out that only pre-transplant sodium levels were considered in these studies. Nevertheless, the found correlations between mortality and liver transplant outcomes may not simply be applicable for kidney transplant recipients.

With most of the existing studies supporting the association between hyponatremia and poorer outcomes in general, hyponatremia is accepted and used as an independent prognostic factor of mortality. 

It must be emphasised that most of the studies that found correlations between hyponatremia and mortality were observational studies and were carried out with an epidemiological rather than a pathophysiological approach. Hence, it is not possible to estimate the amount of possible confounders, which were not taken into account. Hyponatremia may be more a marker of underlying co-morbidities or treatment such as diuretics and its association with mortality may be indirect. 

Moreover, the association to mortality may rely on the causative factor for hyponatremia. Patients with liver disease are often hypervolemic and hyponatremia reflects vasoplegia, whereas hyponatremia in our population may rather be drug or volume depletion related. Causes of hyponatremia, disturbances in water and electrolyte homeostasis respectively, were indeed rarely specified, just as little as the varying medical treatments. Additionally, data was usually not adjusted for the severity of the different comorbidities, which could not only cause hyponatremia but also contribute significantly to patients’ outcomes. 

One study, published in 2011, tried to determine whether patients die from or with hyponatremia [28]: They gathered data of 45,693 hospitalised hyponatremic patients. The clinical course, serum sodium levels from admission till death or hospital release, including its correction rates and comorbidities quantified with the Charlson Comorbidity Score [29], were obtained. They examined the hyponatremic patients who died at their lowest sodium level, and those who survived their sodium low point. Mortality (rates were compared to 164,146 normonatremic patients) increased while serum sodium levels dropped, but only until 120 mmol/L. At lower levels, the mortality rate decreased. Deaths <120 mmol/L were all associated with a significant acute progressive underlying illness, such as acute kidney injury or sepsis, corresponding with a high average Charlson Comorbidity Score of 5.5. On the other hand, among the patients who survived sodium levels <110 mmol/L, severe comorbidities were rare (Charlson Comorbidity Score average 1.8). Hyponatremia was mostly caused by medication and was the main reason for hospital admission. Finally, neurologic complications as a result of hyponatremia were uncommon. These findings support the hypothesis that the extent of illness rather than the severity of hyponatremia itself leads to mortality.

In conclusion, no association between hyponatremia at 6 months post transplantation and decreased renal function, graft loss or mortality, was detected in kidney transplant recipients. Only a secondary analysis found an association between severe hyponatremia and adverse outcome. The strength of our study lies in the study design, as it is embedded in the prospective national Swiss Transplant Cohort Study that comprises six different transplant centers, thus providing a large number of patients and laboratory values. Our study has some limitations: serum sodium concentrations were collected at single time points. Moreover, lipid profile, fluctuating sodium values due to diuretics or other relevant medications as anti-depressant are possible; the group assignment of our study population could therefore be affected. Finally, due to the cohort design of our study, electrolyte and creatinine analysis on routine biochemistry samples was performed in each study center and not centrally.

Nevertheless, our data support the fact that hyponatremia in kidney transplant recipients may not be a crucial prognostic marker and may not designate the most at-risk patients. More studies are needed to assess the possible causal relationship between hyponatremia and mortality as well as the effect of its treatment in different patient’s populations.

## Figures and Tables

**Table 1:** Baseline characteristics of the study population (*n* = 1315): clinical parameters, co-morbidities, transplantation information, outcome.

Parameters	Sodium <136 mmol/l (*n* = 97)	Sodium ≥136 mmol/l (*n* = 1218)	Total (*n* = 1315)
Age at transplantation (years)	56 (48–65)	54 (43–63)	54 (43–63)
Males, *n* (%)	63 (64.95%)	786 (64.53%)	849 (64.56%)
Hypertension, *n* (%)	53 (54.64%)	701 (57.55%)	754 (57.34%)
Liver disease, *n* (%)	1 (1.03%)	10 (0.82%)	11 (0.84%)
Cardiac insufficiency, *n* (%)	19 (19.59%)	204 (16.75%)	223 (16.96%)
**Donor type**			
Donation after Brainstem death (DBD)	64 (65.98%)	629 (51.64%)	693 (52.7%)
Living related	10 (10.31%)	250 (20.53%)	260 (19.77%)
Living unrelated	20 (20.62%)	301 (24.71%)	321 (24.41%)
Donation after Circulatory Death (DCD)	3 (3.09%)	38 (3.12%)	41 (3.12%)
**Dialysis before transplantation**			
Haemodialysis	72 (74.23%)	830 (68.14%)	902 (68.59%)
Peritoneal dialysis	11 (11.34%)	171 (14.04%)	182 (13.84%)
Pre-emptive	14 (14.43%)	217 (17.82%)	231 (17.57%)
**Serum measurements (mean)**			
Sodium (mmol/l)	132.9 ± 3.05	140.3 ± 2.3	140 ± 3.08
Chloride (mmol/l)	102.9 ± 5.17	106.06 ± 6.22	106 ± 6.21
Potassium (mmol/l)	4.21 ± 0.48	4.11 ± 0.48	4.12 ± 0.48
Calcium total (mmol/l)	2.38 ± 0.24	2.39 ± 0.17	2.39 ± 0.17
Phosphate (mmol/l)	0.94 ± 0.21	0.92 ± 0.37	0.92 ± 0.36
Magnesium (mmol/l)	0.7 ± 0.1	0.68 ± 0.1	0.69 ± 0.1
Creatinine (µmol/l)	138.6 ± 60.33	135.26 ± 50.6	136 ± 51.36
Urea (mmol/l)	9.73 ± 5.11	9.98 ± 5.33	9.96 ± 5.32
eGFRCKD-EPI (ml/min/1.73m^2^)	48.22 ± 16.32	48.97 ± 16.03	48.9 ± 16.04
**Outcome**			
Death	4 (4.12%)	52 (4.27%)	56 (4.26%)
Graft loss	3 (3.09%)	24 (1.97%)	27 (2.05%)
Rapid decline of renal function	36 (37.11%)	478 (39.24%)	514 (39.09%)
Composite outcome	43 (44.33%)	554 (45.48%)	597 (45.4%)

**Table 2:** Association of sodium with other electrolytes, blood pressure, age and gender.

	Regression Coefficient	95% CI	*p* Value
Glucose (mmol/L)	−0.25	−0.33–−0.18	<0.001
Chloride (mmol/L)	0.11	0.09–0.14	<0.001
Potassium (mmol/L)	−0.78	−1.09–−0.48	<0.001
Age	0.01	0–0.03	0.0443
Sex (Male)	0.38	0.05–0.71	0.0257
Albumine (g/L)	0.03	0.01–0.05	0.0109
Phosphate (mmol/L)	0.03	−0.35–0.40	0.8902
Magnesium (mmol/L)	0.00	0.0–0.01	0.5586
Systolic blood pressure	0.00	−0.01–0.01	0.4492
Diastolic blood pressure	−0.01	−0.03–0	0.0731
Loop Diuretic	0.1	−0.36–0.55	0.6831
Thiazide Diuretic	0.07	−0.53–0.68	0.8138
Anti-hypertensive (ACE inhibitors, ARB)	−0.08	−0.43–0.28	0.6706
Anti-hypertensive (others)	−0.14	−0.57–0.28	0.5131
Steroids	0.12	−0.29–0.52	0.5801
Mycophénolate mofétil	−0.71	−1.27–−0.15	0.0132
Calcineurin inhibitors	0.6	0.14–1.06	0.0105

## Data Availability

Researchers interested in collaborating with the STCS and accessing data are welcome to submit their proposal to the Scientific Committee of the STCS following the rules laid out in the STCS guidelines and using the templates provided online. https://www.stcs.ch/research/information-for-researchers, accessed on 24 August 2021.

## Supplementary Materials

The following are available online at https://www.mdpi.com/article/10.3390/nu13092995/s1, Figure S1: Kaplan Meier curve of outcome of death by sodium level (<132 mmol/L or ≥132 mmol/L)., Table S1: Impact of serum sodium and relevant outcomes after excluding patients with serum sodium levels >145 mmol/L (n = 21).
