# Supplementary material for: Impact of Hyponatremia after Renal Transplantation on Decline of Renal Function, Graft Loss and Patient Survival: A Prospective Cohort Study
Source: Nutrients. 2021 Aug 27;13(9):2995. doi: 10.3390/nu13092995 (PMC8468476; doi:10.3390/nu13092995)
Supplement: Supplementary file 1 [file nutrients-13-02995-s001.zip › nutrients-1321999-supplementary.pdf]

**Table S1.** Impact of serum sodium and relevant outcomes after excluding patients with serum sodium levels >145 mmol/L (n = 21).

| | | |
|---|---|---|
| **Death**<br>**Normal sodium (≥136 and ≤145 mmol/L)**<br>**Sodium <136 mmol/L** | **Ref**<br>**1.02 (0.47–2.19)** | ***p* = 0.97** |
| Decline of renal function, graft loss or death<br>Normal sodium (≥136 and ≤145 mmol/L)<br>Sodium <136 mmol/L | log rank test | *p* = 0.96 |
| Graft loss or death<br>Normal sodium (≥136 and ≤145 mmol/L)<br>Sodium <136 mmol/L | Ref<br>1.13 (0.63-2.04) | *p* = 0.68 |

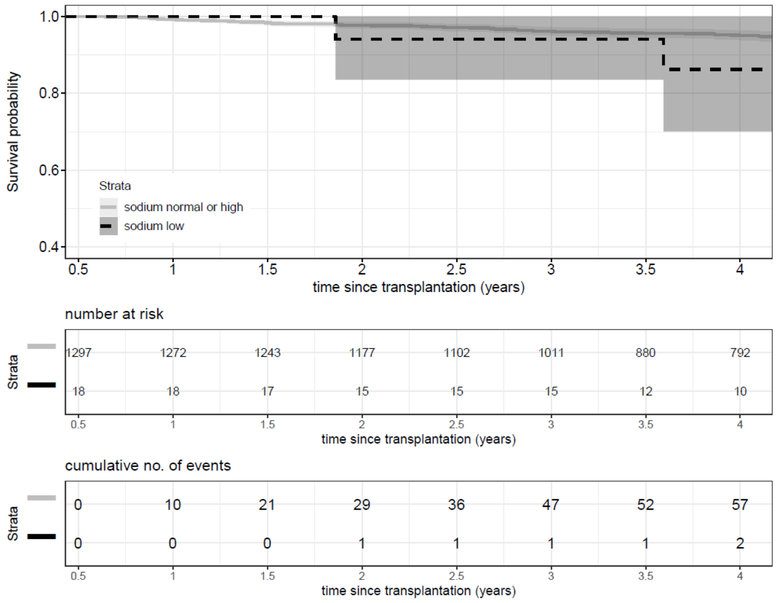


**Figure S1.** Kaplan Meier curve of outcome of death by sodium level (<132 mmol/L or > = 132 mmol/L).
